# Simulations of molecular photodynamics in long timescales

**DOI:** 10.1098/rsta.2020.0382

**Published:** 2022-05-16

**Authors:** Saikat Mukherjee, Max Pinheiro, Baptiste Demoulin, Mario Barbatti

**Affiliations:** ^1^ Aix Marseille University, CNRS, ICR, Marseille, France; ^2^ Institut Universitaire de France, 75231 Paris, France

**Keywords:** theoretical chemistry, excited states, nonadiabatic phenomena, dynamics simulations, photochemistry, computational chemistry

## Abstract

Nonadiabatic dynamics simulations in the long timescale (much longer than 10 ps) are the next challenge in computational photochemistry. This paper delimits the scope of what we expect from methods to run such simulations: they should work in full nuclear dimensionality, be general enough to tackle any type of molecule and not require unrealistic computational resources. We examine the main methodological challenges we should venture to advance the field, including the computational costs of the electronic structure calculations, stability of the integration methods, accuracy of the nonadiabatic dynamics algorithms and software optimization. Based on simulations designed to shed light on each of these issues, we show how machine learning may be a crucial element for long time-scale dynamics, either as a surrogate for electronic structure calculations or aiding the parameterization of model Hamiltonians. We show that conventional methods for integrating classical equations should be adequate to extended simulations up to 1 ns and that surface hopping agrees semiquantitatively with wave packet propagation in the weak-coupling regime. We also describe our optimization of the Newton-X program to reduce computational overheads in data processing and storage.

This article is part of the theme issue ‘Chemistry without the Born–Oppenheimer approximation’.

## 1. Introduction

Simulation of ultrafast molecular photodynamics is a mature field now [1–3]. Computational chemists have an arsenal of methods and research protocols to predict the molecular evolution within a few picoseconds. Nevertheless, most photoinduced processes occur in much longer timescales, several orders of magnitude over our current research capabilities. A few groups, including ours, are already probing dynamics simulations into these long timescales [4–13].

Although the field of *molecular photodynamics in long timescales* is still in the early stages, it should soon undergo an explosive rush of publications, primarily fed by the speed-up allowed by machine learning (ML) techniques [14,15]. However, the challenges are immense and go much beyond computational speeding up. From software optimization to methods' adequacy and algorithmic stability, there are issues in every corner. Thus, the maturation of this research area will require tremendous effort in the following years.

Hence, we decided to write a perspective article about photodynamics in long timescales to help frame the scope of the endeavour ahead. Our group was awarded an ERC Advanced Grant in 2019 precisely to work out methods for this field. Our research since then has provided us with a unique perspective on this subject, which we think is worth sharing in this special issue about *Chemistry without the Born*–*Oppenheimer approximation*.

Before advancing in the discussion, we would like to delimit the scope of this paper by clearing what we expect from a method to run nonadiabatic dynamics in the long timescale. We loosely define ‘long timescales' as periods much longer than 10 ps. Moreover, we believe that the main advantage of dynamics simulations over statistical approaches (like reaction rate calculations) is to propagate the molecular dynamics in full nuclear dimensionality, without bias towards any set of coordinates. Therefore, our first methodological constraint is to focus on mixed quantum classical (MQC) methods [1], which have full dimensionality as the main feature. (All acronyms used in this paper are defined in the electronic supplementary material, SM-1.) Second, we also primarily aim at general methods, which may be used for any molecular system (as opposed, for instance, to a particular potential energy surface fitted for a specific molecule). Finally, long time-scale simulations should not depend upon unrealistically large computational resources, especially if we consider the high economic and environmental costs of high-performance computing [16]. Thus, our third constraint is that those future methods developed to tackle such simulations should ideally allow us to run dynamics without significantly demanding new computational resources.

Having these problem delimitations in mind, we will survey the following topics impacting simulations of excited-state phenomena occurring over long timescales: (i) electronic structure methods speed-up; (ii) stability of integration methods; (iii) accuracy of nonadiabatic dynamics methods; and (iv) software optimization. All this discussion is supported by a series of simulations of adiabatic, surface hopping and quantum wave packet dynamics using a model Hamiltonian, as well as surface hopping and ML modelling of an actual molecule.

## 2. Electronic structure method speed-up

Long time-scale simulations will require a substantial reduction in the cost of quantum mechanical quantities (excited state energies, forces and state couplings) to keep computational resource requirements under control. We envisage three possible strategies to achieve this cost reduction: (i) use of parameterized and approximated electronic structure methods; (ii) use of parameterized full-dimensional Hamiltonian models; and (iii) use of ML as a surrogate model for quantum mechanical predictions. They are discussed in the following subsections.

### (a) Parameterized and approximated electronic structure methods

Parameterized electronic structure methods speed up quantum chemical (QC) calculations by replacing computationally intensive steps like integral calculations with precomputed quantities either based on empirical [17–19] or computational data [20,21]. There are several parameterized electronic structure methods currently available for MQC dynamics, allowing low-cost excited state calculations. Some of them are based on configuration interaction procedures, like FOMO-CI [18] and OM2/CI [17]. In turn, TD-DFTB [20] and the semiempirical TD-SCF [3] compute excited states based on linear-response time-dependent theory.

A different strategy to reduce computational costs without parameterization is met in the real-time time-dependent approach based on Kohn–Sham orbitals (TD-KS) [22], which has also been extensively explored for inexpensive MQC dynamics [23]. TD-KS uses KS orbital energy gaps for approximated excitation energies and estimates forces and nonadiabatic couplings from orbital derivatives.

For decades, parameterized electronic structure methods have been pushing the boundaries of computational photochemistry. The first on-the-fly surface hopping simulations were performed with the semiempirical MMVB method [24]. More recently, nonadiabatic dynamics simulations of the order of 10 ps have been reported with FOMO-CI [18], and nonadiabatic dynamics of nanoscopic systems with hundreds of atoms have been treated with real-time TD-KS based on DFTB orbitals [25].

Nevertheless, parameterized and approximated electronic structure methods usually have low accuracy when considering extended regions of the configurational space [26]. Moreover, for the former methods, the transferability of their parameters to simulate different systems is always a significant issue [27].

The final pressing issue with this strategy for cost reduction is the constrain we proposed in §1: long time-scale dynamics should not require significantly more computational resources than conventional ultrafast dynamics. Therefore, we need to compute excited state properties about 1000 times faster than today to satisfy such a condition. Ground state calculations with parameterized electronic structure methods are swift. DFTB, for instance, has been reported to be 1000 times faster than DFT with a hybrid functional [28]. However, the excited state calculations, based, for instance, on CI procedures, may still pose high costs.

### (b) Parameterized Hamiltonian models

Another strategy to bypass the high costs of solving the quantum electronic problem is constructing a model Hamiltonian that mimics the physical process of interest accurately and meaningfully. Here again, we reiterate that we wish to explore our system in its full nuclear dimensionality inside MQC. In this subsection, we limit our discussion to two types of model Hamiltonians, which can be used to study multi-state nonadiabatic processes by MQC dynamics in full dimensionality, the spin-boson Hamiltonian (SBH) and the vibronic coupling (VC) models.

SBH is a two-state model initially developed for describing dissipative systems [29,30]. However, due to its inherently generic feature and flexibility regarding the number of degrees of freedom and coupling terms, SBH became one of the favourite *toy potentials* to model nonadiabatic phenomena in complex macromolecular systems [31]. The SBH model describes a system with two diabatic electronic states $\left(H_{S}\right)$ that are linearly coupled $\left(H_{SB}\right)$ to a harmonic bath $\left(H_{B}\right)$ consisting of *N* bosons with masses *M_j_*, frequencies, position *R_j_* and momentum *P_j_*. Thus, the full Hamiltonian is

2.1 $$H=H_{S}+H_{B}+H_{SB},$$

where

2.2 $$\begin{array}{rl} H_{S} & \;=\sigma_{z}\varepsilon_{0}-\sigma_{x}\nu_{0},\\ H_{B} & \;={\text{I}}_{2}\frac{1}{2}\sum_{j=1}^{N}\frac{P_{j}^{2}}{M_{j}}+M_{j}\omega_{j}^{2}R_{j}^{2}\\ \;\text{and}\;H_{SB} & \;=\sigma_{z}\sum_{j=1}^{N}g_{j}R_{j}. \end{array}\}$$

In the isolated system, the energy bias and the electronic interstate coupling between the two diabatic electronic states are $\varepsilon_{0}$ and $\nu_{0}$, respectively. $\sigma_{x/y/z}$ are the usual Pauli matrices and ${\text{I}}_{2}$ is the identity matrix of size 2. The coupling constants $\left(g_{i}\right)$ between the system and bath are determined by the spectral density, which characterizes the effect of the bath on transitions between the electronic states. Different types of spectral density have been designed, out of which Ohmic and Debye forms are typically used to study nonadiabatic dynamics. More complex and multi-peaked spectral density can also be constructed by fitting the appropriate experimental data [31,32]. After that, to work with this model, discretization of the coupling constants and frequencies can be carried out by standard protocols [33–35]. SBH can be easily transformed into adiabatic representation [36] to obtain the adiabatic energies, gradients and nonadiabatic coupling vectors (see the electronic supplementary material, SM-2).

Despite being extensively explored in MQC and quantum nonadiabatic dynamics [36–43], SBH remains much needed in the future as a model Hamiltonian to study dynamics in the long timescale. Examples are already discussed below in §3a, where we use SBH to propagate classical dynamics for 1 ns. In §4, we simulated quantum wave packet and surface hopping dynamics with SBH for up to 100 ps. Examples of adiabatic potential energy topographies that SBH can describe are shown in electronic supplementary material, SM-2.

One of the main limitations of the SBH model is its restriction to two electronic states. The VC model developed by Koppel *et al.* [44] (and, for this reason, also known as KDC Hamiltonian) can be an alternative to deal with an arbitrarily large number of states. For $N_{el}$ electronic states, the diabatic Hamiltonian ${\hat{H}}_{vib}$ is decomposed into the kinetic energy of the nuclei $\left({\hat{T}}_{N}\right)$ and harmonic ground state potential energy $\left(V_{0}\right)$ around some reference nuclear geometry (*Q*_0_), using mass-frequency scaled dimensionless normal-mode coordinates (*Q*) and a $N_{el}\times N_{el}$ VC matrix (*W*), which is a low-order Taylor series expansion around the reference nuclear geometry. This prescription renders

2.3 $${\hat{H}}_{\text{vib}}=({\hat{T}}_{N}+V_{0})1+W^{\left(0\right)}+W^{\left(1\right)}+W^{\left(2\right)}+\cdots .$$

The zeroth-order coupling matrix $\left(W^{\left(0\right)}\right)$ is diagonal and contains the adiabatic excitation energies at the reference point *Q*_0_. Truncating the series after the first order leads to the linear VC (LVC) model, which contains the intrastate coupling constant $\left(\kappa_{i}^{n}\right)$ for the $n^{th}$ electronic state and $i^{th}$ mode in the diagonal elements of $W^{\left(1\right)}$ and the interstate coupling constant $\left(\lambda_{i}^{mn}\right)$ between states *m* and *n* in the off-diagonal elements. That means, $W_{nn}^{\left(1\right)}=\sum_{i}\kappa_{i}^{n}Q_{i,}$ and $W_{mn}^{\left(1\right)}=\sum_{i}\lambda_{i}^{mn}Q_{i}.$ Beyond LVC, higher order VC models, including more terms in the Taylor series expansion (equation (2.3)), have also been constructed to describe the PESs [45,46] better and more accurately.

Usually, the interstate coupling parameters are obtained by fitting *ab initio* potential energy surfaces. However, parameters have also been successfully determined using excited state Hessian and wave function overlaps [47–49]. Recently, Plasser *et al*. [50] showed the workability of parameterizing LVC models using only a ground state normal modes calculation and a single-point excited state calculation (including energy gradients) at the equilibrium geometry. Moreover, they proposed a protocol to employ LVC with the standard MQC dynamics methods in adiabatic representation.

It is a common practice to construct the VC model Hamiltonians in reduced dimensionality considering only the important modes for the photophysical process of interest. A 24-dimension quadratic VC model for pyrazine published over 20 years ago still remains an almost solitary example of full-dimensional fitting of a non-trivial molecule [51]. In §1, we stated that we are primarily interested in methods general enough to be applied to any molecule in full nuclear dimensionality. However, the parameterization process is still the main challenge in using VC models for general, full-dimensional dynamics. We include them here because the advances in ML techniques for fitting diabatic surfaces may soon favourably change this scenario, allowing fast model parameterization [45,46].

### (c) ML for excited states

In many MQC dynamics applications, the QC properties of the molecular systems are usually calculated for each time step during the simulation. Such a repeatedly large number of calls to a QC program is the main bottleneck to run long timescale in nonadiabatic dynamics simulations. In this scenario, ML has emerged as a promising approach to speed up the prediction of molecular properties required for excited state molecular dynamics while still retaining the same level of accuracy as the underlying QC method used as a reference to train the ML predictor [14,15].

The main advantage of using ML models as a surrogate for the QC method comes from the fact that, once the ML model has been fitted to the reference data to reproduce QC properties within the desired accuracy, the solution of the Schrödinger equation is wholly skipped, thereby resulting in extremely fast predictions for new molecular configurations produced during the dynamics. This type of multi-dimensional regression problem is known as supervised learning in the ML field, where kernel methods and neural networks have become the preferred algorithms for fitting (highly nonlinear) chemical data [52].

However, performing fully ML-driven excited state dynamics is rather challenging [14,15]. One crucial issue is related to the intrinsic multi-dimensional nature of the target properties required for an MQC simulation. The ML model should accurately predict the potential energy of the diverse molecular configurations accessible in each electronic state (which are scalar properties) and interatomic forces and nonadiabatic coupling vectors (vectorial quantities). Also, the ML model should preserve possible correlations between the predicted variables (e.g. interatomic forces are proportional to the energy gradients), so that the ML-driven dynamics still obey certain physical principles such as energy conservation. Finally, the accuracy of the ML model is strictly related to the sampling strategy adopted to generate the training dataset, which should include the most representative molecular configurations accessible during the time evolution of the system on different excited-state surfaces.

Despite these challenges, the highly efficient computational performance of various ML models has been successfully demonstrated for some prototypical photoreactive molecules [9,13,53–55]. In some of these previous studies, it was possible to achieve a nanosecond timescale in the nonadiabatic dynamics, although the molecules investigated undergo decay processes towards the ground state in a much shorter timescale, typically hundreds of femtoseconds. To our knowledge, the potentialities of ML to run nonadiabatic dynamics for more complex systems with intrinsically slow photorelaxation processes such as in fluorescence remains to be explored.

Notably, the most advanced ML models designed for predicting potential energy surfaces already meet the chemical accuracy threshold (1.0 kcal mol^−1^ = 0.043 eV) [56]. However, this accuracy level depends on the complexity of the molecule and the availability of QC forces to better interpolate the potential energy surfaces in the training process. Moreover, a common problem found in the ML-PES literature is that the models' accuracy concerning energy predictions tends to degrade when going from the ground state to high-order electronic states [53]. This effect can be attributed to the more complex nature and higher diffuseness of the electronic density in excited states, as well as the dense packing of electronic states whose mixing may result in non-smooth PES for the excited states [1].

To illustrate some of the difficulties in learning excited state energies, we computed a dataset of 50 surface hopping trajectories for protonated 7-azaindole at the ADC(2)/cc-pVDZ [57] level (electronic supplementary material, SM-3) to train a kernel-ridge regression model with the RE descriptor, the so-called KREG model [58] (electronic supplementary material, SM-4). The discussion of the physical–chemical properties of this system will be reported elsewhere. Here, we focus exclusively on how well ML can predict its energies.

**Figure 1.**
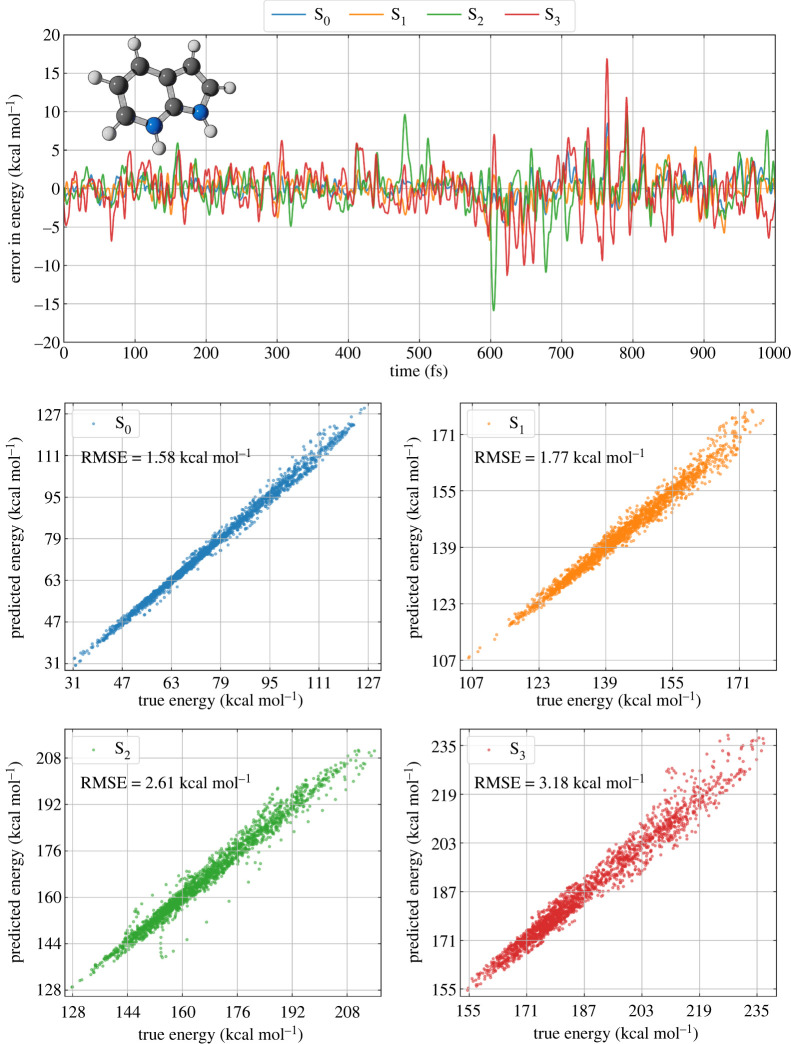
Performance of the KREG model for predicting the ground- and excited-state energies of an entire MD trajectory (test set) of protonated 7-azaindole. (Online version in colour.)

By plotting the difference between the ML-predicted and the ADC(2) energies as a function of time for each state (figure 1, top panel), one can see that the errors vary approximately in the range of −5 to 5 kcal mol^−1^ for all states along the first half of the testing trajectory. However, the disagreement between QC energies and ML predictions increases significantly after 600 fs, especially for S_2_ and S_3_. This inaccuracy is reflected in the RMSE, which increases by a factor of two when going from the ground state to the highest excited state S_3_ (see the bottom panels in figure 1). Note that the energy deviation $\left(|E_{KREG}-E_{QC}|\right)$ for some specific geometries can be as large as 15 kcal mol^−1^ when the molecule is in S_2_ or S_3_ even though the corresponding RMSE values (averaged over the whole trajectory) are much smaller (2.61 and 3.18 kcal mol^−1^, respectively). The consequences of such a significant error for a quantitative description of the photodynamics of molecules with ML are a critical issue to be addressed in future studies.

Photochemical reactions in molecular systems are often characterized by multiple decay channels or pathways that may end up with considerably different photoproducts. Within the surface hopping approach, this behaviour can be translated into the proportion of ‘typical’ trajectories representing a particular set of electronic features or molecular geometries in dynamics evolution. Some of these ‘typical’ trajectories may be described as rare events because they represent only a tiny fraction of the total number of simulated trajectories. From the perspective of ML, this discussion raises a central question when developing robust and accurate models for nonadiabatic dynamics: the sampling strategy used to build the training set. Since ML is grounded on statistical principles, the sample of molecular geometries used as a training set should represent as close as possible the distribution of the entire (in principle, unknown) population. However, the unbalanced distribution of ‘typical’ trajectories and molecular configurations often found in nonadiabatic dynamics pose a challenge for constructing high-quality training sets.

**Figure 2.**
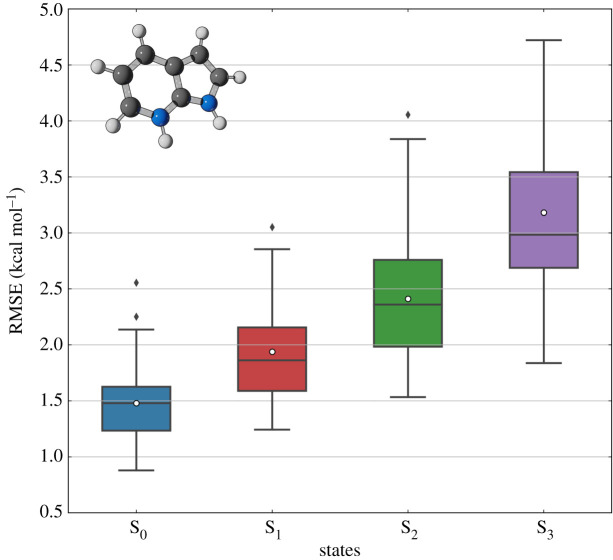
Assessment of KREG model uncertainty for predicting ground- and excited-state energies of an entire surface hopping trajectory of protonated 7-azaindole. Each boxplot contains the distribution of RMSE values of energy predictions for one entire trajectory (test set) out of 50 independent MD simulations. The model was trained on 5000 molecular geometries randomly sampled from 49 trajectories after separating one trajectory for testing. This process was repeated until each one of the 50 MD trajectories were evaluated. The white dots in the boxplots represent the mean value of the RMSE distribution, while black dots indicate the outliers. (Online version in colour.)

The boxplots of figure 2, representing the RMSE distribution for each state of the protonated 7-azaindole dataset, sheds light on this problem. This statistical distribution is obtained using a ‘leave-one-out’ cross-validation scheme in the trajectory space, meaning that one trajectory is selected as the test set for each round of training and evaluation (electronic supplementary material, SM-4). As shown in figure 2, the RMSE values are spread in a wide range, and they increase when moving to high-order excited states (approximately 1.3 kcal mol^−1^ in S_0_ to 2.9 kcal mol^−1^ in S_3_). A few trajectories appear as outliers in the error distribution (see the black dots in figure 2). These findings indicate that geometries belonging to atypical trajectories are probably underrepresented in the training set. Different sampling strategies [59,60] have been proposed to remedy this issue and improve the quality of the training set, with active learning [61–63] being the most used in nonadiabatic dynamics applications.

## 3. Stability of integration methods

Even if we manage to substantially bring computational costs down enough to allow the long time-scale propagation, the simulation results must be reliable. Unfortunately, however, long trajectories mean more error accumulation. In the frame of classical nuclear motions, these errors are of two types, first coming from the integration of Newton's equations; second, coming from the zero-point energy (ZPE) spilling. We discuss them in the next subsections.

### (a) Errors in the integration methods

Long time-scale simulations put enormous stress on the integration algorithms. Longer trajectories require smaller time steps to reduce error accumulation [64] but at the expense of increased computational costs. This subsection assesses how the velocity Verlet algorithm [65], which is often employed to integrate the classical equation of motions (EOM), behaves in the long timescale.

For our tests, we employed the SBH model discussed in §2b. The model was set with 33 dimensions, equivalent to a molecule with 13 atoms (see details in electronic supplementary material, SM-2). We have run four trajectories of 1 ns each, to test different aspects. All trajectories started with the same initial conditions and were set as microcanonical ensembles. Because our goal is to check the stability of the classical EOM, the simulations were limited to adiabatic dynamics in state 2. The set-up of each trajectory is:

— Trajectory 1: time step 0.01 fs. This trajectory is taken as the benchmark.
— Trajectory 2: time step 0.5 fs. It has the largest time step acceptable for integration of the classical EOMs.
— Trajectory 3: time step 0.5 fs plus random fluctuation of $10^{-7}r$ Hartree/Bohr added to the SBH adiabatic forces, where *r* is a uniform random number between 0 and 1.
— Trajectory 4: time step 0.5 fs plus random fluctuation of $10^{-5}r$ Hartree/Bohr.

The random fluctuations in trajectories 3 and 4 aim at assessing how sensitive the integration is to the precision of the electronic structure calculations. Precision plays a role whenever there is a chance that the electronic structure method may yield a different result when an identical run is repeated. This problem occurs in nonlinear systems converging to different local solutions due to tiny underlying numerical fluctuations [66]. In dynamics, it expresses as small, unphysical discontinuities on the potential energy surface.

**Figure 3.**
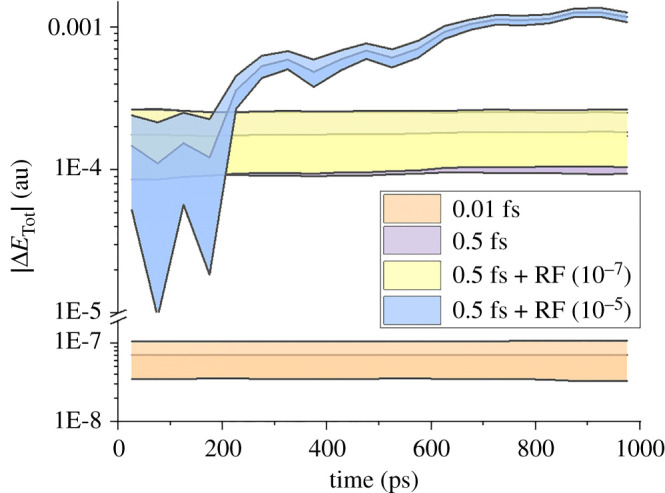
Distribution of total energy deviation as a function of time for different integration set-ups. Each dataset shows the mean (central curve) plus and minus one s.d. (shaded area) for points collected in intervals of 50 ps. Each set-up is characterized by the time step (0.01 or 0.5 fs), and random fluctuation (RF) added to the forces, 10^−7^ or 10^−5^ Hartree/Bohr. (Online version in colour.)

Figure 3 shows the total energy conservation level in each trajectory in terms of the absolute total energy variation at time *t*, $|\Delta E_{\text{Tot}}|=|\Delta E_{\text{Tot}}\text{( }t\text{) }-E_{\text{Tot}}\text{( 0)}|$. The entire trajectory was sliced in intervals of 50 ps. For each interval, we computed the mean and standard deviation of $|\Delta E_{\mathrm{Tot}}|$. Unsurprisingly, with a tiny time step like Δ*t* = 0.01 fs, an excellent level of energy conservation is obtained, with absolute variations of (7 ± 4) × 10^−8^ Hartree for the entire range up to 1 ns. With Δ*t* = 0.5 fs, the variation increases significantly to (2 ± 1) × 10^−4^ Hartree. On a positive note, this level of variation remains constant during the entire trajectory, dissipating our initial fear of data degradation at long times. Random fluctuations of about 10^−7^ Hartree/Bohr do not impact the trajectory. However, random fluctuations 100 times larger, of about 10^−5^ Hartree/Bohr, degrade the long time-scale energy conservation, with absolute variations reaching a dangerous level of (1.0 ± 0.1) × 10^−3^ Hartree at 1 ns.

### (b) ZPE spilling

ZPE is a consequence of the quantum uncertainty principle, and thereby, an inherent error creeps into the propagation of classical trajectories in MQC methods by neglecting it. The classical nuclear motion approximation permits reactions below the quantum threshold of energy leading to unphysical results where products can be formed with less energy than ZPE in the internal vibration degrees of freedom. The problem becomes serious if the ZPE flows out of several modes and pumps into a specific weak bond to make it unphysically hot.

This ZPE spilling phenomenon is well discussed in the literature [67–70]. Nevertheless, it has been systematically neglected in simulations of ultrafast dynamics because of the assumption that it takes longer than the photochemical process to kick in. Whether this hypothesis is justified or not, we obviously cannot lean on that anymore when simulating dynamics in the long timescale.

Different strategies have been designed to reinforce ZPE maintenance during quasi-classical trajectories [71–79]. Most of them [71–75] require calculating instantaneous normal modes and Hessians, which may be cumbersome to be applied in on-the-fly dynamics propagation. In turn, Xie & Bowman proposed [80] a method that, in principle, can be applied to the on-the-fly dynamics of a general polyatomic system. Nevertheless, it has not yet been tested in this context.

Apart from the above discussed active ZPE constraint models, a few passive techniques have been suggested to overcome the ZPE leaking problem in classical trajectory calculations [76–79]. These models discard unphysical trajectories from the statistics that either form products with less than their ZPE or form products where the sum of the product vibrational energies is less than the total product ZPE. The drawback is that passive techniques may bias the results if any of the competing reaction pathways is more prone to undergo ZPE leaking problems than the others.

At this point, we agree with the conclusion of Guo *et al*. [69] that no satisfactory solution to the infamous ZPE spilling problem in classical dynamics has been found to date. Thus, novel techniques tailored to on-the-fly dynamics must be developed if we aim at long time-scale dynamics [81].

## 4. Accuracy of nonadiabatic dynamics

Suppose we managed to reduce computational costs and control the nuclear integration errors in the long time-scale propagation. Are the MQC methods available today tailored to deal with nonadiabatic phenomena in the long timescale? For instance, can surface hopping reasonably predict hops in weakly coupled regions?

This section demonstrates our first attempt to address these questions by investigating the performance of the decoherence-corrected fewest-switches surface hopping (DC-FSSH) dynamics in the long timescale. DC-FSSH results are compared with full-quantum results calculated with multi-configurational time-dependent Hartree (MCTDH) [82,83] and its multi-layer variant ML-MCTDH [84–86]. The tests are based on the SBH model discussed in §2b with 10 harmonic bath modes and Debye spectral density, constructed to describe the nonadiabaticity in the weak coupling limit [87] (see the electronic supplementary material, SM-2).

Methodologies for long timescale, nonadiabatic dynamics in high-nuclear dimensionality are so underdeveloped that we cannot rely on any method as an absolute benchmark. Even the state-of-the-art MCTDH cannot be ensured to perform well in such an unprobed domain. Bearing such limitations in mind, we performed long time-scale dynamics aiming to check whether the results yielded by the different methods are consistent between them. Given that MCTDH is a full-quantum methodology while DC-FSSH is a mixed quantum-classical ad hoc approach [88], we assume without proving that the former should still deliver the most accurate result.

The MCTDH ansatz expands the nuclear wave function into time-dependent single-particle functions (SPFs), where the latter are expressed as a linear combination of time-independent primitive basis. It immediately reduces the number of basis functions required for converged calculation by providing the variationally determined basis for an optimal description of the evolving wave packet. The Dirac–Frenkel variational principle is used to derive coupled nonlinear differential equations as the MCTDH EOM, usually solved by the predictor–corrector integrator scheme.

ML-MCTDH is an extension of the MCTDH approach, in which the multi-dimensional SPFs are propagated themselves within the MCTDH ansatz. The standard propagation method describes a single layer of expansion coefficients for the time-independent basis. By contrast, MCTDH contains a first layer of expansion coefficients for the time-dependent SPF basis and the second layer of time-dependent expansion coefficients that parameterize the time evolution of the SPFs. One can expand the multi-dimensional time-dependent SPFs again using an MCTDH ansatz yielding a three-layer scheme. In other words, the ML-MCTDH method prescribes to apply the MCTDH scheme successively in different layers to propagate the SPFs.

The Heidelberg MCTDH package is used to simulate MCTDH and ML-MCTDH [89,90] dynamics on the 10-dimensional SBH model system. Here, we limit our discussion by presenting only the results keeping the number of SPFs n=16 and the grid size N=64. Details of the MCTDH and ML-MCTDH set-ups are given in the electronic supplementary material, SM-5.

Trajectory surface hopping [1,91] is a widely known MQC dynamics methodology, which expresses the nuclear wave packet on a particular electronic surface as a swarm of independent classical trajectories obeying Newton's equations, and the force acting on the nuclei is derived as the negative gradient of the potential energy of the corresponding electronic state. At each time step, a stochastic algorithm determines whether the system will propagate on the current electronic state or hop to another one leading to nonadiabatic population transfer between the electronic states. Among various strategies to compute transition probability, Tully's FSSH formulation is probably the most common and extensively reviewed in the literature [92].

We have used Newton-X NS (novel series; see §5) to carry out the DC-FSSH dynamics (electronic supplementary material, SM-6) on the SBH model in the adiabatic representation. Since we performed the DC-FSSH in the adiabatic representation and MCTDH in the diabatic representation, comparing the electronic population obtained from these two methodologies would be unjustified. Thereby, as Landry *et al*. [39] prescribed, we have calculated the diabatic electronic populations from a swarm of DC-FSSH trajectories using an orthogonal adiabatic to diabatic transformation matrix and mixing angles [93–95]. The diabatic population decay curves are fitted with a bi-exponential function to obtain the time constants $\left(\tau_{1}\;\text{and}\;\tau_{2}\right)$ associated with the nonadiabatic process,

4.1 $$f(t)=N_{0}+(1-N_{0})\left[(1-A)\exp\left(-\frac{t}{\tau_{1}}\right)+A\exp\left(-\frac{t}{\tau_{2}}\right)\right],$$

where $N_{0}$ is the remaining population that does not decay in these timescales. Since the bi-exponential function could not fit the population decay curve obtained from the MCTDH dynamics, the following mono-exponential function is also used to fit only the MCTDH population curve

4.2 $$f_{\text{mono}}(t)=N_{0}+(1-N_{0})\exp\left(-\frac{t}{\tau_{1}}\right).$$

**Figure 4.**
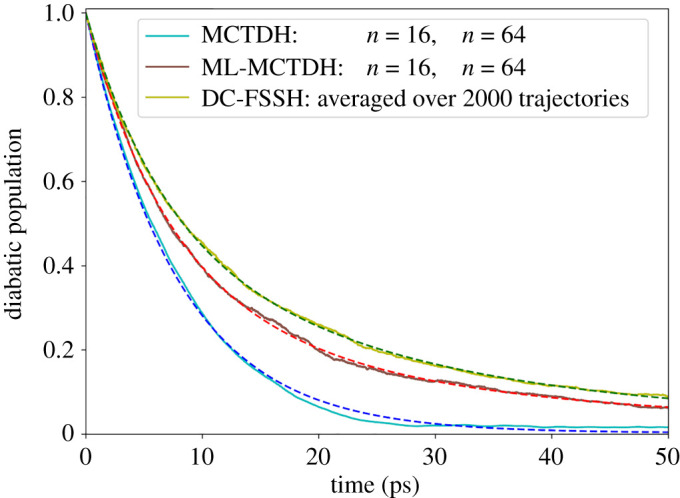
The diabatic population decay of the excited state for different dynamics methodologies are shown in solid lines, and the corresponding fitted curves are presented in dashed lines. (Online version in colour.)

ML-MCTDH and DC-FSSH yield closely matching time constants, predicting similar kinds of dynamics undergoing with two different timescales (figure 4 and table 1). However, it is surprising to see that, unlike the multi-layer variant, the MCTDH population curve has a single time constant. The long time constant is due to a repopulation of the excited state rather than to a state trapping. We could not yet determine why it shows up in ML-MCTDH but not in MCTDH. We are proceeding with the analysis running new simulations, but discussing these results is out of the scope of this paper. It will require a separate publication exclusively dedicated to this subject. Despite these differences, it can be safely stated that the calculated time constants of the non-radiative relaxation process obtained from quantum dynamics and MQC dynamics are of the same order of magnitude, validating the latter method even in the long timescale.

**Table 1.** Fitted parameters for the diabatic populations in the long time-scale (100 ps) dynamics of an SBH model. *A* and $\tau_{2}$ are not defined for MCTDH.

	*A*	$\tau_{1}$ (ps)	$\tau_{2}$ (ps)	*N*_0_
MCTDH	—	7.85	—	0.003
ML-MCTDH	0.34	7.07	23.71	0.024
DC-FSSH	0.50	6.45	23.31	0.028

## 5. Software optimization

Originally, Newton-X, our software program for dynamics simulation on excited states, was developed in the frame of ultrafast processes [96,97]. For this reason, in the choice between computational efficiency and flexibility (modularity to communicate with different electronic structure methods), most of the time, the latter was favoured. Moreover, it is not fit for processing and storing big data. Simulations in the long timescale invert the priorities. Therefore, efficiency and big data become mandatory issues to be addressed.

We have recently started to write a completely new Newton-X version to deal with these issues. The legacy program is now referred to as Newton-X CS (for classical series), while the new program is referred to as Newton-X NS (for novel series). We will present the details of the Newton-X NS implementation in a future publication, but here, we would like to highlight a few points about how it will help move towards long time-scale simulations.

From a technical standpoint, performing nonadiabatic dynamics with FSSH amounts to repeating the following sequence:

(i) Read initial conditions (geometry, velocity, initial electronic state and initial time-dependent wave function coefficients).
(ii) Compute electronic energies and energy gradients (usually through an external code).
(iii) Obtain time-derivative couplings (either nonadiabatic coupling vectors, through wave function overlap matrices, or approximated methods [98]).
(iv) Solve the classical EOM.
(v) Solve the quantum EOM (a locally approximated time-dependent Schrödinger equation).
(vi) Decide on whether to hop to another state or not.
(vii) Go back to step (i) with the updated set of conditions.

Going between steps requires conveying information either internally (from (v) to (vi), for instance) or through some external program ((i) to (ii), for example). A straightforward approach is to use a primary driver that will call a set of scripts or programs sequentially, each communicating through text files. Although this approach maximizes modularity, it has clear limits in terms of I/O operations.

Historically, step (ii) has been the time-limiting step in on-the-fly dynamics simulations, as performing electronic structure computations is much more resource-intensive than the EOM integration. Indeed, most operations in steps (iv) and (v) scale with the number of atoms or the number of states included in the system, while step (ii) scales with a power of the number of electrons, making the I/O operations effectively costless. With the development of fast electronic structure calculations highlighted in §2, this relation does not hold anymore, and the dynamics approach must be adapted to limit the number of I/O operations. This algorithmic restructuring is especially crucial for long time-scale dynamics, where we must tightly optimize the speed of each step. Replacing a set of scripts with a more integrated program can solve this issue by keeping most data in memory during the dynamics and only writing on disk at specific points when unavoidable, like when communicating with an external program.

To deal with this problem, we have entirely restructured Newton-X. While Newton-X CS is a collection of Perl scripts with Fortran programs, Newton-X NS is a complete Fortran program using Perl only for dealing with QM input files. To show the performance of the Newton-X NS, we considered a 1 ps dynamics using the 10D SBH model (electronic supplementary material, SM-2), integrated with a time step of 0.1 fs (resulting in 10 000 simulation steps). In Newton-X CS, such a trajectory takes 20 400 s (5 h 40 min) to complete, while Newton-X NS computes it in only 11 s, astonishingly almost 1850 times faster. Of course, the increase in performance when interfaced with external programs would be less impressive, as the time for the electronic structure computation is likely to be dominant in this case.

The second software-related problem we must deal with when planning for long time-scale dynamics is data storage. Molecular dynamics simulations output a gargantuan amount of data, as the outcome is primarily a long sequence of geometries and energies. This problem is even more pronounced in FSSH, as the algorithm's stochastic nature requires an ensemble of many trajectories. We can make a simple estimate of what these data requirements would represent in the long timescale. Let us assume we performed simulations for a molecule with 20 atoms for a 1 ns trajectory with 0.5 fs time step. The storage of the geometries alone (in double precision) would require around 1 GB of space. Standard FSSH studies routinely need around 100 trajectories to achieve satisfying statistical accuracy, so the geometries alone could take up to 100 GB of space. To this amount, we should still add electronic populations, energies, nonadiabatic couplings and surface hopping-related data (like the transition probabilities). This dataset must be further post-processed to deliver physical–chemical information and made publicly available to comply with open data requirements. It is thus of crucial importance that the data files are uniquely identified and that the set-up for producing the data (software version, operating system, computer used) is also available.

The current state in Newton-X CS is to output these data as a series of text files. Although convenient to write and read, text files have some serious drawbacks. For each new dataset, a new file must be created and documented. This can quickly lead to a multiplication of outputs, making data integrity harder to ensure and data sharing more difficult. Therefore, obtaining the output as a single file containing the entire dataset produced, as well as the condition of production as metadata, would be a desirable feature. This can be achieved with a structured file format. However, the context of MQC dynamics makes the use of structured text formats cumbersome.

In MQC dynamics, we save data as time series. In practice, this means that the effective rank of each dataset is increased by one. Although conventional formats exist for saving two-dimensional data like molecular geometries as a time series (*xyz* format, for instance), keeping track of data with higher rank, such as energy gradients when several electronic states are involved, quickly becomes cumbersome and decreases the readability associated with text files. Another problem lies in the analysis of trajectories. Each time step will produce heterogeneous data, which must be saved when they are still available in memory, but for convenience of analysis, it is desirable that the data corresponding to the different sets (energies, populations, …) are kept together. Structured text formats exist, like JSON or YAML, but they are not suited for a sequential update of the datasets. One solution would be to save the time steps as a parent field, containing the heterogeneous data as children, post-process the file at the end of the simulation to gather all datasets and produce a new structured file with consistent datasets.

These issues can be solved using the hierarchical data format (HDF) model, which allows the creation of portable, self-described and open binary file formats. The model has clear advantages [99], with complete C, C++ and Fortran APIs, and officially supported bindings with Python, Julia and R. The files created with the underlying library can be easily explored and shared and are self-documented, with the inclusion of metadata directly along with the datasets. Binary files also occupy less disk space than text files, and HDF5 can add compression to the data to lower the footprint even more. We have chosen to adopt the H5MD format [100]—specifically designed to handle molecular dynamics—to Newton-X NS. Using this file format allows us to benefit from all the environments already developed.

For the same simulation as the one showed earlier in this section, set to write the maximum amount of output and using Newton-X NS, the program spends around 3–4 s to write output files, showing no difference between text and H5MD files. However, the files obtained differ in size: the text files together occupy 58 MB of disk space, while the H5MD file occupies only 28 MB. It is also worth noticing that HDF5 files can be read in chunks, thus facilitating the treatment of large datasets, and can be opened only partially just to read a header, creating exciting opportunities for automated archiving and research in databases.

## 6. Conclusion

Nonadiabatic dynamics simulations in the long timescale (defined here as much longer than 10 ps) have been an overlooked research area. Several recent works in the field indicate that this situation may be about to change. In this paper, we lay down the elements that should be considered for advancing the research in this field.

First, we delimit the scope of what we expect from methods to run such long time-scale simulations: they should work in full nuclear dimensionality, be general enough to tackle any type of molecular system and be computationally affordable. Bearing these constraints in mind, we examined the main methodological challenges we should face to advance the field: the computational costs of the electronic structure calculations, the stability of the integration methods, the accuracy of the nonadiabatic dynamics algorithms and software optimization. We discuss each of these issues based on simulations of model systems and actual molecules.

We analysed three strategies to speed up electronic structure calculations, using parameterized and approximated methods, employing model Hamiltonians and predicting electronic properties with ML. The first strategy is consistently associated with strong accuracy loss. The second has the main challenge of parameterizing full-dimensional systems, a procedure that has found an ally in ML. For the third strategy, we believe ML may be on the verge of becoming a crucial element for long time-scale dynamics. Nevertheless, it still misses clear protocols for training excited-state energies of large conformational sets and high density of states with the accuracy needed for dynamics.

We showed that conventional methods for integrating the classical EOM simulations should be adequate to extended simulations up to 1 ns, as long as the precision of the electronic structure properties is kept under control. Nevertheless, ZPE leaking must be accounted for. Hessian-free methods for correcting ZPE leaking need to be developed.

We evaluated the performance of decoherence-corrected surface hopping in the long timescale. The results for internal conversion in the weak-coupling regime show that it can make predictions in semiquantitative agreement with wave packet propagation.

Finally, we also described our optimization of the Newton-X program to reduce computational overheads in processing and data storage. In the most favourable case, computational time can be reduced more than 1000 times compared with the costs of simulations done on the current software architecture.

## Data Availability

The following datasets are available: 7-AIH^+^ dataset [101]; 33-D SBH trajectories [102]; 10-D SBH DC-FSSH trajectories [103] and quantum wave packets [104].

## References

[1] Crespo-Otero R, Barbatti M. 2018 Recent advances and perspectives on nonadiabatic mixed quantum-classical dynamics. Chem. Rev. **118**, 7026-7068. (10.1021/acs.chemrev.7b00577) 29767966

[2] Curchod BF, Martínez TJ. 2018 Ab initio nonadiabatic quantum molecular dynamics. Chem. Rev. **118**, 3305-3336. (10.1021/acs.chemrev.7b00423) 29465231

[3] Nelson TR *et al.* 2020 Non-adiabatic excited-state molecular dynamics: theory and applications for modeling photophysics in extended molecular materials. Chem. Rev. **120**, 2215-2287. (10.1021/acs.chemrev.9b00447) 32040312

[4] Vacher M, Mendive-Tapia D, Bearpark MJ, Robb MA. 2014 The second-order Ehrenfest method. Theor. Chem. Acc. **133**, 1505. (10.1007/s00214-014-1505-6)

[5] Lingerfelt DB, Williams-Young DB, Petrone A, Li X. 2016 Direct ab initio (meta-)surface-hopping dynamics. J. Chem. Theory Comput. **12**, 935-945. (10.1021/acs.jctc.5b00697) 26855086

[6] Nijamudheen A, Akimov AV. 2017 Excited-state dynamics in two-dimensional heterostructures: SiR/TiO_2_ and GeR/TiO_2_ (R = H, Me) as promising photocatalysts. J. Phys. Chem. C **121**, 6520-6532. (10.1021/acs.jpcc.7b00545)

[7] Akimov AV. 2017 Stochastic and quasi-stochastic Hamiltonians for long-time nonadiabatic molecular dynamics. J. Phys. Chem. Lett. **8**, 5190-5195. (10.1021/acs.jpclett.7b02185) 28985075

[8] Barbatti M. 2020 Simulation of excitation by sunlight in mixed quantum-classical dynamics. J. Chem. Theory Comput. **16**, 4849-4856. (10.1021/acs.jctc.0c00501) 32579345 PMC7426902

[9] Westermayr J, Gastegger M, Menger MFSJ, Mai S, González L, Marquetand P. 2019 Machine learning enables long time scale molecular photodynamics simulations. Chem. Sci. **10**, 8100-8107. (10.1039/C9SC01742A) 31857878 PMC6849489

[10] Jingbai L, Patrick R, André E, Pascal F, Steven L. 2020 Nanosecond photodynamics simulations of a cis-trans isomerization are enabled by machine learning. ChemRxiv. (10.26434/chemrxiv.13047863)

[11] Lan J, Kapil V, Gasparotto P, Ceriotti M, Iannuzzi M, Rybkin VV. 2021 Simulating the ghost: quantum dynamics of the solvated electron. Nat. Commun. **12**, 766. (10.1038/s41467-021-20914-0) 33536410 PMC7859219

[12] Akimov AV. 2021 Extending the time scales of nonadiabatic molecular dynamics via machine learning in the time domain. J. Phys. Chem. Lett. **12**, 12 119-12 128. (10.1021/acs.jpclett.1c03823) 34913701

[13] Li J, Reiser P, Boswell BR, Eberhard A, Burns NZ, Friederich P, Lopez SA. 2021 Automatic discovery of photoisomerization mechanisms with nanosecond machine learning photodynamics simulations. Chem. Sci. **12**, 5302-5314. (10.1039/D0SC05610C) 34163763 PMC8179587

[14] Dral PO, Barbatti M. 2021 Molecular excited states through a machine learning lens. Nat. Rev. Chem. **5**, 388-405. (10.1038/s41570-021-00278-1) 37118026

[15] Westermayr J, Marquetand P. 2020 Machine learning for electronically excited states of molecules. Chem. Rev. **5**, 388-405. (10.1021/acs.chemrev.1020c00749) PMC8391943 33211478

[16] Stevens ARH, Bellstedt S, Elahi PJ, Murphy MT. 2020 The imperative to reduce carbon emissions in astronomy. Nat. Astron. **4**, 843-851. (10.1038/s41550-020-1169-1)

[17] Korth M, Thiel W. 2011 Benchmarking semiempirical methods for thermochemistry, kinetics, and noncovalent interactions: OMx methods are almost as accurate and robust as DFT-GGA methods for organic molecules. J. Chem. Theory Comput. **7**, 2929-2936. (10.1021/ct200434a) 26605482

[18] Accomasso D, Granucci G, Wibowo M, Persico M. 2020 Delocalization effects in singlet fission: comparing models with two and three interacting molecules. J. Chem. Phys. **152**, 244125. (10.1063/5.0009914) 32610952

[19] Granucci G, Toniolo A. 2000 Molecular gradients for semiempirical CI wavefunctions with floating occupation molecular orbitals. Chem. Phys. Lett. **325**, 79-85. (10.1016/S0009-2614(00)00691-6)

[20] Kranz JJ, Elstner M, Aradi B, Frauenheim T, Lutsker V, Garcia AD, Niehaus TA. 2017 Time-dependent extension of the long-range corrected density functional based tight-binding method. J. Chem. Theory Comput. **13**, 1737-1747. (10.1021/acs.jctc.6b01243) 28272887

[21] Domínguez A, Aradi B, Frauenheim T, Lutsker V, Niehaus TA. 2013 Extensions of the time-dependent density functional based tight-binding approach. J. Chem. Theory Comput. **9**, 4901-4914. (10.1021/ct400123t) 26583409

[22] Akimov AV, Prezhdo OV. 2013 The PYXAID program for non-adiabatic molecular dynamics in condensed matter systems. J. Chem. Theory Comput. **9**, 4959-4972. (10.1021/ct400641n) 26583414

[23] Wang L, Long R, Prezhdo OV. 2015 Time-domain ab initio modeling of photoinduced dynamics at nanoscale interfaces. Annu. Rev. Phys. Chem. **66**, 549-579. (10.1146/annurev-physchem-040214-121359) 25622188

[24] Smith BR, Bearpark MJ, Robb MA, Bernardi F, Olivucci M. 1995 Classical wavepacket dynamics through a conical intersection - application to the S_1_/S_0_ photochemistry of benzene. Chem. Phys. Lett. **242**, 27-32. (10.1016/0009-2614(95)00718-J)

[25] Pal S, Trivedi DJ, Akimov AV, Aradi B, Frauenheim T, Prezhdo OV. 2016 Nonadiabatic molecular dynamics for thousand atom systems: a tight-binding approach toward PYXAID. J. Chem. Theory Comput. **12**, 1436-1448. (10.1021/acs.jctc.5b01231) 26954907

[26] Maitra NT. 2006 On correlated electron-nuclear dynamics using time-dependent density functional theory. J. Chem. Phys. **125**, 014110. (10.1063/1.2210471) 16863290

[27] Dral PO, von Lilienfeld OA, Thiel W. 2015 Machine learning of parameters for accurate semiempirical quantum chemical calculations. J. Chem. Theory Comput. **11**, 2120-2125. (10.1021/acs.jctc.5b00141) 26146493 PMC4479612

[28] Elstner M, Seifert G. 2014 Density functional tight binding. Phil. Trans. R. Soc. A **372**, 20120483. (10.1098/rsta.2012.0483) 24516180

[29] Leggett AJ, Chakravarty S, Dorsey AT, Fisher MPA, Garg A, Zwerger W. 1987 Dynamics of the dissipative two-state system. Rev. Mod. Phys. **59**, 1-85. (10.1103/RevModPhys.59.1)

[30] Weiss U. 1999 Quantum dissipative systems. Singapore: World Scientific.

[31] Mendive-Tapia D, Mangaud E, Firmino T, de la Lande A, Desouter-Lecomte M, Meyer H-D, Gatti F. 2018 Multidimensional quantum mechanical modeling of electron transfer and electronic coherence in plant cryptochromes: the role of initial bath conditions. J. Phys. Chem. B **122**, 126-136. (10.1021/acs.jpcb.7b10412) 29216421

[32] Tamura H, Martinazzo R, Ruckenbauer M, Burghardt I. 2012 Quantum dynamics of ultrafast charge transfer at an oligothiophene-fullerene heterojunction. J. Chem. Phys. **137**, 540. (10.1063/1.4751486) 23249077

[33] Makri N. 1999 The linear response approximation and its lowest order corrections: an influence functional approach. J. Phys. Chem. B **103**, 2823-2829. (10.1021/jp9847540)

[34] Kelly A, Markland TE. 2013 Efficient and accurate surface hopping for long time nonadiabatic quantum dynamics. J. Chem. Phys. **139**, 014104. (10.1063/1.4812355) 23822290

[35] Wang H, Song X, Chandler D, Miller WH. 1999 Semiclassical study of electronically nonadiabatic dynamics in the condensed-phase: spin-boson problem with Debye spectral density. J. Chem. Phys. **110**, 4828-4840. (10.1063/1.478388)

[36] Chen H-T, Reichman DR. 2016 On the accuracy of surface hopping dynamics in condensed phase non-adiabatic problems. J. Chem. Phys. **144**, 094104. (10.1063/1.4942867) 26957154

[37] Wang H, Thoss M. 2008 From coherent motion to localization: dynamics of the spin-boson model at zero temperature. New J. Phys. **10**, 115005. (10.1088/1367-2630/10/11/115005)

[38] Tempelaar R, Reichman DR. 2017 Generalization of fewest-switches surface hopping for coherences. J. Chem. Phys. **148**, 102309. (10.1063/1.5000843) 29544318

[39] Landry BR, Falk MJ, Subotnik JE. 2013 Communication: the correct interpretation of surface hopping trajectories: how to calculate electronic properties. J. Chem. Phys. **139**, 211101. (10.1063/1.4837795) 24320356

[40] Jain A, Subotnik JE. 2015 Surface hopping, transition state theory, and decoherence. II. Thermal rate constants and detailed balance. J. Chem. Phys. **143**, 134107. (10.1063/1.4930549) 26450292

[41] Hughes KH, Christ CD, Burghardt I. 2009 Effective-mode representation of non-Markovian dynamics: a hierarchical approximation of the spectral density. II. Application to environment-induced nonadiabatic dynamics. J. Chem. Phys. **131**, 124108. (10.1063/1.3226343) 19791853

[42] Mac Kernan D, Ciccotti G, Kapral R. 2002 Surface-hopping dynamics of a spin-boson system. J. Chem. Phys. **116**, 2346-2353. (10.1063/1.1433502)

[43] Ben-Nun M, Martínez TJ. 2007 A continuous spawning method for nonadiabatic dynamics and validation for the zero-temperature spin-boson problem. Isr. J. Chem. **47**, 75-88. (10.1560/IJC.47.1.75)

[44] Koppel H, Domcke W, Cederbaum LS. 1984 Multimode molecular-dynamics beyond the Born–Oppenheimer approximation. Adv. Chem. Phys. **57**, 59-246. (10.1002/9780470142813.ch2)

[45] Williams DMG, Eisfeld W. 2018 Neural network diabatization: a new ansatz for accurate high-dimensional coupled potential energy surfaces. J. Chem. Phys. **149**, 204106. (10.1063/1.5053664) 30501255

[46] Shen Y, Yarkony DR. 2020 Construction of quasi-diabatic hamiltonians that accurately represent ab initio determined adiabatic electronic states coupled by conical intersections for systems on the order of 15 atoms. Application to cyclopentoxide photoelectron detachment in the full 39 degrees of freedom. J. Phys. Chem. A **124**, 4539-4548. (10.1021/acs.jpca.0c02763) 32374600

[47] Lévêque C, Komainda A, Taïeb R, Köppel H. 2013 Ab initio quantum study of the photodynamics and absorption spectrum for the coupled 11A2 and 11B1 states of SO_2_. J. Chem. Phys. **138**, 044320. (10.1063/1.4776758) 23387597

[48] Schuurman MS, Yarkony DR. 2007 On the vibronic coupling approximation: a generally applicable approach for determining fully quadratic quasidiabatic coupled electronic state Hamiltonians. J. Chem. Phys. **127**, 094104. (10.1063/1.2756540) 17824729

[49] Plasser F, Ruckenbauer M, Mai S, Oppel M, Marquetand P, González L. 2016 Efficient and flexible computation of many-electron wave function overlaps. J. Chem. Theory Comput. **12**, 1207-1219. (10.1021/acs.jctc.5b01148) 26854874 PMC4785508

[50] Plasser F, Gómez S, Menger MFSJ, Mai S, González L. 2019 Highly efficient surface hopping dynamics using a linear vibronic coupling model. Phys. Chem. Chem. Phys. **21**, 57-69. (10.1039/C8CP05662E) 30306987

[51] Raab A, Worth GA, Meyer HD, Cederbaum LS. 1998 Molecular dynamics of pyrazine after excitation to the S2 electronic state using a realistic 24-mode model Hamiltonian. J. Chem. Phys. **110**, 936-946. (10.1063/1.478061)

[52] Unke OT, Chmiela S, Sauceda HE, Gastegger M, Poltavsky I, Schütt KT, Tkatchenko A, Müller K-R. 2021 Machine learning force fields. Chem. Rev. **121**, 10 142-10 186 (10.1021/acs.chemrev.0c01111) PMC8391964 33705118

[53] Hu D, Xie Y, Li X, Li L, Lan Z. 2018 Inclusion of machine learning kernel ridge regression potential energy surfaces in on-the-fly nonadiabatic molecular dynamics simulation. J. Phys. Chem. Lett. **9**, 2725-2732. (10.1021/acs.jpclett.8b00684) 29732893

[54] Chen W-K, Liu X-Y, Fang W-H, Dral PO, Cui G. 2018 Deep learning for nonadiabatic excited-state dynamics. J. Phys. Chem. Lett. **9**, 6702-6708. (10.1021/acs.jpclett.8b03026) 30403870

[55] Westermayr J, Faber FA, Christensen AS, von Lilienfeld OA, Marquetand P. 2020 Neural networks and kernel ridge regression for excited states dynamics of CH_2_NH_2_^+^: from single-state to multi-state representations and multi-property machine learning models. Mach. Learn. Sci. Technol. **1**, 025009. (10.1088/2632-2153/ab88d0)

[56] Unke OT, Meuwly M. 2019 PhysNet: a neural network for predicting energies, forces, dipole moments, and partial charges. J. Chem. Theory Comput. **15**, 3678-3693. (10.1021/acs.jctc.9b00181) 31042390

[57] Dreuw A, Wormit M. 2015 The algebraic diagrammatic construction scheme for the polarization propagator for the calculation of excited states. WIREs: Comp. Mol. Sci. **5**, 82-95. (10.1002/wcms.1206)

[58] Dral PO, Ge F, Xue B-X, Hou Y-F, Pinheiro M, Huang J, Barbatti M. 2021 MLatom 2: an integrative platform for atomistic machine learning. Top. Curr. Chem. **379**, 27. (10.1007/s41061-021-00339-5) PMC8187220 34101036

[59] Dral PO, Owens A, Yurchenko SN, Thiel W. 2017 Structure-based sampling and self-correcting machine learning for accurate calculations of potential energy surfaces and vibrational levels. J. Chem. Phys. **146**, 244108. (10.1063/1.4989536) 28668062

[60] Fonseca G, Poltavsky I, Vassilev-Galindo V, Tkatchenko A. 2021 Improving molecular force fields across configurational space by combining supervised and unsupervised machine learning. J. Chem. Phys. **154**, 124102. (10.1063/5.0035530) 33810678

[61] Botu V, Ramprasad R. 2015 Adaptive machine learning framework to accelerate ab initio molecular dynamics. Int. J. Quantum Chem. **115**, 1074-1083. (10.1002/qua.24836)

[62] Smith JS, Nebgen B, Lubbers N, Isayev O, Roitberg AE. 2018 Less is more: sampling chemical space with active learning. J. Chem. Phys. **148**, 241733. (10.1063/1.5023802) 29960353

[63] Imbalzano G, Zhuang Y, Kapil V, Rossi K, Engel EA, Grasselli F, Ceriotti M. 2021 Uncertainty estimation for molecular dynamics and sampling. J. Chem. Phys. **154**, 074102. (10.1063/5.0036522) 33607885

[64] Odell A, Delin A, Johansson B, Cawkwell MJ, Niklasson AMN. 2011 Geometric integration in born-oppenheimer molecular dynamics. J. Chem. Phys. **135**, 224105. (10.1063/1.3660689) 22168678

[65] Swope WC, Andersen HC, Berens PH, Wilson KR. 1982 A computer-simulation method for the calculation of equilibrium-constants for the formation of physical clusters of molecules - application to small water clusters. J. Chem. Phys. **76**, 637-649. (10.1063/1.442716)

[66] Hurd P, Cusati T, Persico M. 2010 Trajectory integration with potential energy discontinuities. J. Comput. Phys. **229**, 2109-2116. (10.1016/j.jcp.2009.11.025)

[67] Wu Sf, Marcus R. 1970 Analytical mechanics of chemical reactions. V. Application to the linear reactive H + H2 systems. J. Chem. Phys. **53**, 4026-4035. (10.1063/1.1673874)

[68] Truhlar DG, Kuppermann A. 1970 Quantum mechanics of the H + H2 reaction: exact scattering probabilities for collinear collisions. J. Chem. Phys. **52**, 3841-3843. (10.1063/1.1673570)

[69] Guo Y, Thompson DL, Sewell TD. 1996 Analysis of the zero-point energy problem in classical trajectory simulations. J. Chem. Phys. **104**, 576-582. (10.1063/1.470853)

[70] Wang H, Peslherbe GH, Hase WL. 1994 Trajectory studies of SN2 nucleophilic substitution. 4. Intramolecular and unimolecular dynamics of the Cl—CH_3_Br and ClCH_3_—Br-complexes. J. Am. Chem. Soc. **116**, 9644-9651. (10.1021/ja00100a032)

[71] Bowman JM, Gazdy B, Sun Q. 1989 A method to constrain vibrational energy in quasiclassical trajectory calculations. J. Chem. Phys. **91**, 2859-2862. (10.1063/1.456955)

[72] Miller WH, Hase WL, Darling CL. 1989 A simple model for correcting the zero point energy problem in classical trajectory simulations of polyatomic molecules. J. Chem. Phys. **91**, 2863-2868. (10.1063/1.456956)

[73] Lim KF, McCormack DA. 1995 The conservation of quantum zero-point energies in classical trajectory simulations. J. Chem. Phys. **102**, 1705-1715. (10.1063/1.468697)

[74] Bonhommeau D, Truhlar DG. 2008 Mixed quantum/classical investigation of the photodissociation of NH_3_(*Ã*) and a practical method for maintaining zero-point energy in classical trajectories. J. Chem. Phys. **129**, 014302. (10.1063/1.2943213) 18624475

[75] Varandas AJC, Marques JMC. 1994 Method for quasiclassical trajectory calculations on potential energy surfaces defined from gradients and Hessians, and model to constrain the energy in vibrational modes. J. Chem. Phys. **100**, 1908-1920. (10.1063/1.466544)

[76] Townsend D, Lahankar SA, Lee SK, Chambreau SD, Suits AG, Zhang X, Rheinecker J, Harding LB, Bowman JM. 2004 The roaming atom: straying from the reaction path in formaldehyde decomposition. Science **306**, 1158-1161. (10.1126/science.1104386) 15498970

[77] Shepler BC, Braams BJ, Bowman JM. 2007 Quasiclassical trajectory calculations of acetaldehyde dissociation on a global potential energy surface indicate significant non-transition state dynamics. J. Phys. Chem. A **111**, 8282-8285. (10.1021/jp074646q) 17676724

[78] Kurosaki Y. 2006 Energy-flow dynamics in the molecular channel of propanal photodissociation, C_2_H_5_CHO → C_2_H_6_ + CO: direct ab initio molecular dynamics study. J. Phys. Chem. A **110**, 11 230-11 236. (10.1021/jp063452s) 17004731

[79] Shepler BC, Braams BJ, Bowman JM. 2008 ‘Roaming’ Dynamics in CH_3_CHO photodissociation revealed on a global potential energy surface. J. Phys. Chem. A **112**, 9344-9351. (10.1021/jp802331t) 18597443

[80] Xie Z, Bowman JM. 2006 Zero-point energy constraint in quasi-classical trajectory calculations. J. Phys. Chem. A **110**, 5446-5449. (10.1021/jp055861e) 16623473

[81] Mukherjee S, Mario B. 2022 A Hessian free method to prevent zero-point energy leakage in classical trajectory simulation. ChemRxiv. (10.26434/chemrxiv-2022-53g43) 35679615

[82] Beck MH, Jäckle A, Worth GA, Meyer HD. 2000 The multiconfiguration time-dependent Hartree (MCTDH) method: a highly efficient algorithm for propagating wavepackets. Phys. Rep. **324**, 1-105. (10.1016/S0370-1573(99)00047-2)

[83] Meyer H-D. 2012 Studying molecular quantum dynamics with the multiconfiguration time-dependent Hartree method. WIREs: Comput. Mol. Sci. **2**, 351-374. (10.1002/wcms.87)

[84] Vendrell O, Meyer H-D. 2011 Multilayer multiconfiguration time-dependent Hartree method: implementation and applications to a Henon–Heiles Hamiltonian and to pyrazine. J. Chem. Phys. **134**, 044135. (10.1063/1.3535541) 21280715

[85] Wang H, Thoss M. 2003 Multilayer formulation of the multiconfiguration time-dependent Hartree theory. J. Chem. Phys. **119**, 1289-1299. (10.1063/1.1580111)

[86] Manthe U. 2008 A multilayer multiconfigurational time-dependent Hartree approach for quantum dynamics on general potential energy surfaces. J. Chem. Phys. **128**, 164116. (10.1063/1.2902982) 18447430

[87] Englman R, Jortner J. 1970 The energy gap law for radiationless transitions in large molecules. Mol. Phys. **18**, 145-164. (10.1080/00268977000100171)

[88] Subotnik JE, Ouyang W, Landry BR. 2013 Can we derive Tully's surface-hopping algorithm from the semiclassical quantum Liouville equation? Almost, but only with decoherence. J. Chem. Phys. **139**, 214107. (10.1063/1.4829856) 24320364

[89] Worth GA, Beck MH, Jäckle A, Meyer HD. 2019 The MCTDH Package, Used Version: 8.4.18. See http://Mctdh.Uni-Hd.De.

[90] Worth GA, Beck MH, Jäckle A, Meyer HD. 2019 The MCTDH Package, Used Version: 8.5.10. See http://Mctdh.Uni-Hd.De.

[91] Tully JC. 1990 Molecular-dynamics with electronic-transitions. J. Chem. Phys. **93**, 1061-1071. (10.1063/1.459170)

[92] Granucci G, Persico M. 2007 Critical appraisal of the fewest switches algorithm for surface hopping. J. Chem. Phys. **126**, 134114. (10.1063/1.2715585) 17430023

[93] Baer M. 2006 Beyond born-oppenheimer: electronic nonadiabatic coupling terms and conical intersections. Hoboken, NJ: John Wiley & Sons.

[94] Köppel H. 2011 Conical intersections - theory, computation and experiment. In Diabatic represeantation: methods for construction of diabatic electronic states (eds W Domcke, DR Yarkony, H Köppel), pp. 175-204. Singapore: World Scientific.

[95] Naskar K, Mukherjee S, Mukherjee B, Ravi S, Mukherjee S, Sardar S, Adhikari S. 2020 ADT: a generalized algorithm and program for beyond Born–Oppenheimer equations of ‘*N*’ dimensional sub-Hilbert space. J. Chem. Theory Comput. **16**, 1666-1680. (10.1021/acs.jctc.9b00948) 32003993

[96] Barbatti M, Ruckenbauer M, Plasser F, Pittner J, Granucci G, Persico M, Lischka H. 2014 Newton-X: a surface-hopping program for nonadiabatic molecular dynamics. WIREs: Comp. Mol. Sci. **4**, 26-33. (10.1002/wcms.1158)

[97] Barbatti M, Granucci G, Persico M, Ruckenbauer M, Vazdar M, Eckert-Maksić M, Lischka H. 2007 The on-the-fly surface-hopping program system Newton-X: application to ab initio simulation of the nonadiabatic photodynamics of benchmark systems. J. Photochem. Photobiol. A **190**, 228-240. (10.1016/j.jphotochem.2006.12.008)

[98] do Casal MT, Toldo J, Pinheiro Jr M, & Barbatti M. 2021 Fewest switches surface hopping with Baeck-an couplings [version 1; peer review: 3 approved]. Open Res. Eur. **1**, 49. (10.12688/openreseurope.13624.1) PMC10446015 37645211

[99] Poinot M. 2010 Five good reasons to use the hierarchical data format. Comput. Sci. Eng. **12**, 84-90. (10.1109/MCSE.2010.107)

[100] de Buyl P, Colberg PH, Höfling F. 2014 H5MD: a structured, efficient, and portable file format for molecular data. Comput. Phys. Commun. **185**, 1546-1553. (10.1016/j.cpc.2014.01.018)

[101] Pinheiro Jr M, Mukherjee S, Barbatti M. 2021 Cartesian coordinates of 7-AIH^+^ [Dataset]. (10.6084/m9.figshare.14823126)

[102] Pinheiro Jr M, Mukherjee S, Barbatti M. 2021 SBH trajectories data [Dataset]. (10.6084/m9.figshare.14873094)

[103] Mukherjee S. 2021 DC-FSSH trajectory for 10D SBH mode [Dataset]. Zenodo. (10.5281/zenodo.5039522)

[104] Saikat M. 2021 MCTDH and ML-MCTDH simulation Data for SBH model [Dataset]. (10.6084/m9.figshare.14869089)

